# Hexakis-2-(β-carboxyethenylphenoxy)cyclotriphosphazene: Synthesis, Properties, Modeling Structure

**DOI:** 10.3390/molecules28186571

**Published:** 2023-09-11

**Authors:** Pavel Yudaev, Anastasia Konstantinova, Vladimir Volkov, Evgeniy Chistyakov

**Affiliations:** 1 Mendeleev University of Chemical Technology of Russia, Miusskaya sq., 9, 125047 Moscow, Russia; 2 Shubnikov Institute of Crystallography, Federal Scientific Research Centre “Crystallography and Photonics”, Russian Academy of Sciences, Leninsky Prospect, 59, 119333 Moscow, Russia

**Keywords:** phosphazene, modeling structure, nanoparticle, quantum-chemical calculation, thermal crystallization, heat-resistant polymer

## Abstract

Condensation of hexakis-2-(formylphenoxy)cyclotriphosphazene with malonic acid yielded hexakis-2-(β-carboxyethenylphenoxy)cyclotriphosphazene (2-CEPP), whose structure was confirmed by ^31^P, ^1^H, ^13^C NMR spectroscopy and MALDI-TOF mass spectrometry. A quantum-chemical calculation for the 2-CEPP molecule using the ab initio methods in the 6-311G^**^ basis set and the DFT-PBE0/6-311g** method was performed with geometry optimization of all parameters by the standard gradient method. The acid strength of 2-CEPP was theoretically estimated. Using the small-angle X-ray scattering method, it was found that 2-CEPP is an amorphous substance, which, when heated, can transform into a crystalline state. However, when heated at 370 °C, 2-CEPP undergoes decarboxylation and polymerization to form an insoluble heat-resistant product. The occurrence of decarboxylation and polymerization reactions in the formed styrene fragments was confirmed by thermal (differential-scanning calorimetry) and spectral (solid-state ^13^C NMR spectroscopy) analysis.

## 1. Introduction

Due to the increasing demand for modern materials with improved performance, interest in organic–inorganic cyclic systems and polymers is growing. Among them, cyclic compounds containing phosphorus and nitrogen [1,2,3,4,5]; phosphorus and oxygen [6]; boron and oxygen [7,8]; nitrogen, oxygen, and silicon [9] atoms; nitrogen-containing heterocyclic compounds based on imidazole and guanidine [10], including proton sponges, linear siloxanes, and phosphazenes [11,12,13]; organotin polymers [14]; and copper-containing coordination polymers [15] should be noted. Researchers continue to find new applications for such compounds. For example, oxazadisilinane compounds are precursors for obtaining protic ionic liquids that act as catalysts and solvents for the esterification reaction of acetic acid [9]. The incorporation of methacrylate-containing silsesquioxane–siloxane oligomers into dental polymer compositions improves their mechanical characteristics [16].

Organoboron fluorescent conjugated polymers, such as poly (3-aminophenyl boronic acid), are used to manufacture fluorescent sensors that make it possible to quickly and with high sensitivity determine trace amounts of toxic benzoyl peroxide in wheat flour and antimicrobial agent samples [17].

Particular attention is paid to cyclophosphazenes and compounds based on them, since they are nonflammable, chemically stable, thermally stable, bioinert, and also able to maintain elasticity at low temperatures. Cyclophosphazenes are ideal objects for obtaining various organic high molecular weight compounds, as well as dendrimers and metal complexes on their basis [18,19,20,21,22].

Addition of 5 wt.% hexaglycidylcyclotriphosphazene to a 4,4′-methylenedianiline cured bisphenol A (DHEBA) epoxy resin improves the mechanical properties of the resin due to its increased flexural modulus and hardness and its electrical properties [23]. Cyclotriphosphazenes increase the fire resistance of polymers. For example, epoxy resin based on diglycidyl ether of bisphenol A (DGEBA) cured with hexacyclohexylaminocyclotriphosphazene and diaminotetracyclohexylaminocyclotriphosphazene showed a lower heat release rate compared with the resin cured with ethylenediamine [24]. Addition of 5 wt.% star-shaped cyclotriphosphazene with allyl groups to bismaleimide resin suppresses smoke and heat release during the combustion of the resin, decreasing total heat release by 43% [25]. The authors explain this fact by the condensation and gas phase flame-retardant effects of cyclotriphosphazene.

Bisphenol-A-based benzoxazines contained in the composition of cyclotriphosphazene fragments are able to polymerize at a lower temperature than those in free-phosphazene analogues. In addition, phosphazene-containing polybenzoxazines have increased tensile strength [26] and thermal stability, as well as reduced flammability [27].

Introduction of a 5 wt.% phenoxyphosphazene oligomer into a polyimide film based on hydrogenated 3,3′,4,4′-biphenyltetracarboxylic dianhydride and 4,4′-oxydianiline provides the film with nonflammable features [28].

Phosphazenes are successfully used in medicine [29,30,31,32]; due to the high affinity of phosphorous and nitrogen for dental tissues, they have good affinity for dental tissue and non-toxicity, have little effect on the viscosity of their compositions, and significantly improve the performance of the resulting products, which determines their use in dentistry [29].

Cyclophosphazenes, due to their biocompatibility, act as precursors for the synthesis of biologically active compounds used in pharmaceuticals. Di and tetra dipeptide substituted spiro cyclotriphosphazenes have a cytotoxic effect on cancer cells of the human breast, ovaries, prostate, and colon [33]. Chalkone-substituted cyclotriphosphazenes reduce the viability of human ovarian and prostate cancer cells by 70–90% [34,35]. Cytotoxic activity is due to damage to the DNA of cancer cells.

An important area of research is the preparation of organophosphazenes containing carboxyl groups in their composition [36]. Modifiers of dental compositions [29], materials for microencapsulation [37], hydrogels [38], heat-resistant and non-combustible resins [18,39,40], hemocompatible films [41], and vaccine adjuvants [42,43] were obtained on their basis.

A great prospect for the use of carboxyl-containing aryloxycyclophosphazenes, in our opinion, is their use as modifiers of epoxy resins. This is due to the potential ability of such phosphazenes to act as resin hardeners and, thereby, improve the performance properties of the resulting materials. However, most carboxyl-containing phosphazenes are not compatible with resins due to their structural features and large numbers of hydrogen bonds. Therefore, the goal of this work was to synthesize a carboxyl-containing phosphazene, hexakis-2-(β-carboxyethenylphenoxy)cyclotriphosphazene (2-CEPP), with a new molecular architecture, and study it, which is necessary to evaluate the potential use of the compound as an epoxy resin modifier.

## 2. Results and Discussion

**Figure 1:**
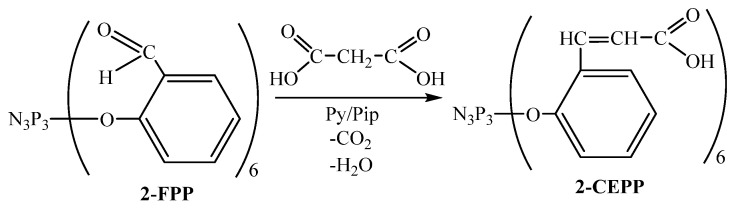
Synthesis scheme for 2-CEPP.

2-CEPP was obtained from the reaction of hexakis-2-(formylphenoxy)cyclotriphosphazene (2-FPP) with malonic acid in refluxing pyridine according to the Knoevenagel–Doebner method [44] as shown in Figure 1.

Piperidine was used as a catalyst. The end of the process was determined by the absence of bubbles of carbon dioxide released during the reaction. The product was isolated by pouring the reaction mixture into hydrochloric acid to remove the solvent, catalyst, and also decomposition salts of 2-CEPP.

**Figure 2:**
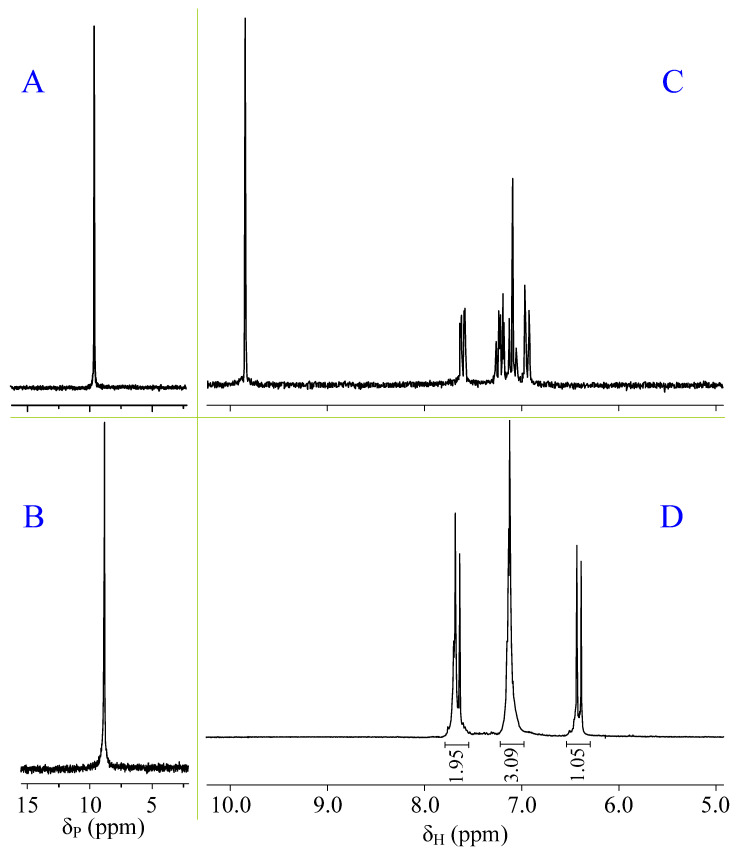
^31^P NMR spectra: 2-FPP (**A**) and 2-CEFF (**B**), ^1^H NMR spectra: 2-FPP (**C**) and 2-CEFF (**D**).

The structure of the obtained compound and the completeness of condensation was confirmed by NMR spectroscopy. The phosphorous spectrum of the reaction product contains a singlet signal in the region of 9 ppm (Figure 2B), which indicates the preservation of the phosphazene cycle during the synthesis. In addition, when comparing the spectrum of the product with the spectrum of 2-FPP (Figure 2A), one can notice a difference in the chemical shifts of signals by 0.8 ppm, which is due to the influence of long-range orders of substituents on the phosphazene ring and indirectly confirms the transformation of aldehyde groups into others.

**Figure 3:**
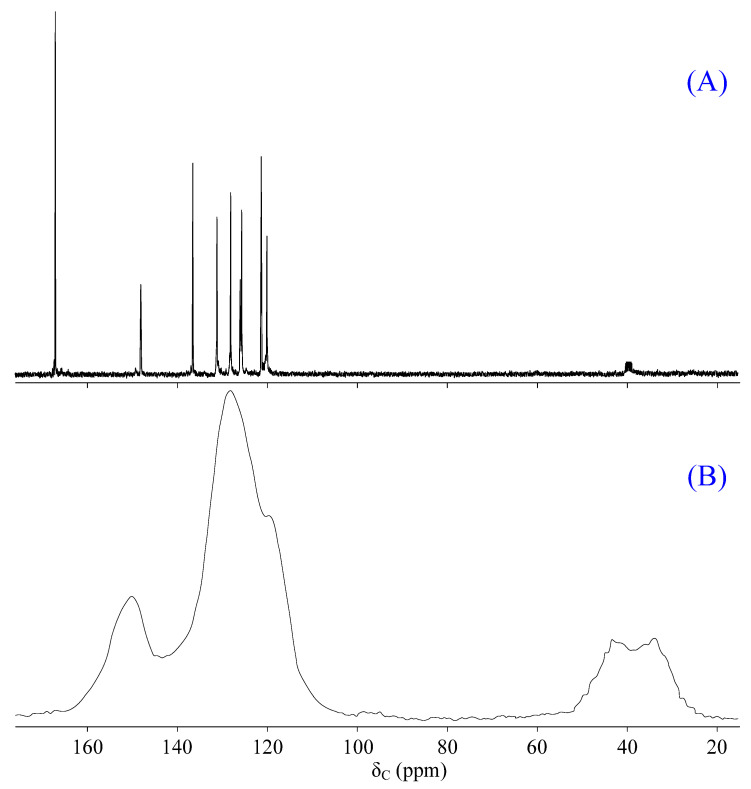
Solution ^13^C NMR spectrum of the initial 2-CEPP (**A**) and solid-state ^13^C NMR spectrum of 2-CEPP heated at 370 °C (**B**).

This fact is confirmed by proton spectroscopy. Comparing the spectra of 2-FPP (Figure 2C) and the obtained product (Figure 2D) shows that the proton signal of the aldehyde group (9.8 ppm) is absent in the spectrum of the product. At the same time, it is not possible to establish the formation of carboxyl groups from the proton spectrum due to the possible deuterium exchange between carboxyl groups and the deuterated solvent (DMSO-*d_6_*). Therefore, it was decided to additionally carry out ^13^C NMR spectroscopy of the product, due to which the formation of carboxyl groups was confirmed, the characteristic signal of carbon atoms of which was observed in the region of 168 ppm (Figure 3A). However, in quantitative terms, their content in the carbon spectrum cannot be estimated.

**Figure 4:**
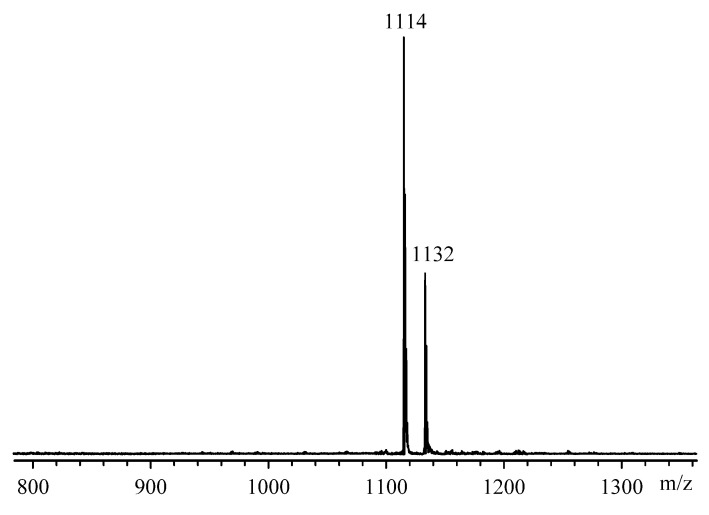
MALDI-TOFF mass spectra for 2-CEFF.

The formation of the target product was finally confirmed by MALDI-TOF mass spectrometry (Figure 4). The spectrum contains the signal of a molecular ion [M + H]^+^ at 1114 *m*/*z* corresponding to 2-CEPP and a minor peak with m/z 1132 belonging to 2-CEPP solvated by a water molecule.

Energy-dispersive X-ray spectroscopy was used to determine the composition of the obtained compound. Based on the results obtained (Table 1), the atomic and weight contents of elements in the sample correspond to the theoretically calculated ones, which confirms the formation of 2-CEFF.

2-CEPP proved to be soluble in aqueous alkaline solutions (pH 9.5), highly polar aprotic solvents (DMF, DMSO), and various diamines (isophorondiamine, ethylenediamine), and when heated in glycols (glycerol, ethylene glycol), but insoluble in non-polar solvents and many others, such as acetone, tetrahydrofuran (THF), and water.

It has been suggested that poor solubility is due to hydrogen bonds, which are broken by solvating solvents and bases as well as heating in high-boiling alcohols.

To confirm the hypothesis, a quantum-chemical calculation for the 2-CEPP molecule was carried out using the ab initio and DFT methods. Calculations by both methods give similar results. The optimized geometric and electronic structure of the phosphazene molecule is shown in Figure S1.

The sphere diameter of the 2-CEPP molecule, namely the distance between the H(43) and H(62) atoms, was 17.06 Å for the ab initio method and 17.15 Å for the DFT method, respectively. These values make it possible to attribute the 2-CEPP molecule to nanosized particles. As a result of the calculation, it was also found that there are intramolecular hydrogen bonds in the 2-CEPP molecule: for the ab initio method—O(42)-H(100) = 1.96 Å, O(61)-H(119) = 1.98 Å; for the DFT method—O(42)-H(100) = 1.84 Å, O(61)-H(119) = 1.84 Å.

In addition, the solubility of acids in polar solvents can be affected by their ability to dissociate. Therefore, the acid strength of 2-CEPP was also theoretically evaluated. The value of the universal acidity index was calculated using the following formulas:

Ab initio/6-311G**: pK_a_ = 49.04–134.61q_max_H^+^ = 9 (qmaxH^+^ = +0.298 is the maximum charge on the hydrogen atom, see Supplementary Materials, Table S1).

DFT-PBE0/6-311g**: pK_a_ = 51.048–150.078q_max_H^+^ = 11 (q_max_H^+^ = +0.269, see Supplementary Materials, Table S2).

Thus, we can conclude that the 2-CEPP molecule belongs to the class of weak acids (9 < pK_a_ < 14), which is consistent with the fact that the substance is insoluble in water, but soluble in aqueous alkaline solutions.

**Figure 5:**
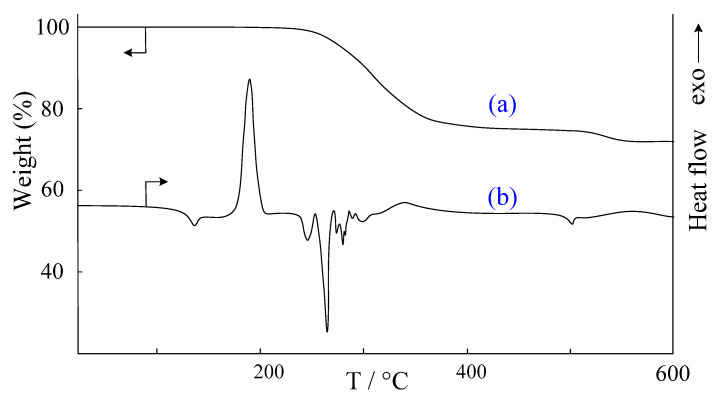
TGA (**a**) and DSC (**b**) curves of 2-CEPP.

For use as a hardener modifier of epoxy resins, phosphazene must be well soluble in them. However, at room temperature, 2-CEPP turned out to be insoluble in epoxy resins, which is due to the presence of hydrogen bonds. These bonds can be broken thermally by melting the substance. However, it should be taken into account that phosphazene must melt at temperatures lower than the curing and/or decomposition temperature of the epoxy resins. Therefore, in order to identify potential applications for 2-CEPP, its thermal behavior was studied. The DSC thermogram of phosphazene shows a jump in heat capacity at 130 °C, which indicates the devitrification of the substance and the presence of an amorphous phase in it (Figure 5b). However, it is impossible to conclude from DSC whether phosphazene is completely amorphous or partially crystalline, because, after the jump in heat capacity, an exothermic peak is observed with a maximum at 185 °C, rather than an endothermic peak characteristic of crystal melting.

**Figure 6:**
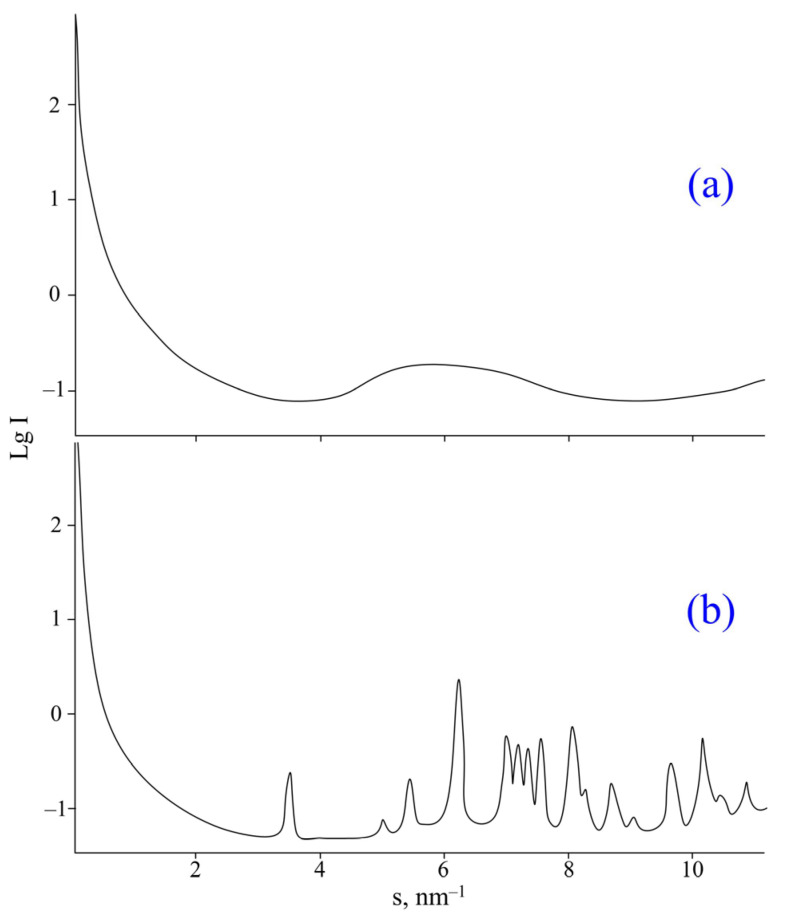
X-ray diffraction pattern of 2-CEPP (**a**) and 2-CEPP heated at 200 °C (**b**).

This substance has been extensively studied with the small-angle scattering method. On the X-ray diffraction pattern of the sample (Figure 6a) there are no peaks characteristic of crystalline substances, producing a halo characteristic of completely amorphous substances.

However, the nature of the exothermic peak at 185 °C remains unclear. Since no weight loss was observed according to TGA (Figure 6a), it is unlikely that the exothermic effect is due to any chemical reaction. Therefore, it was assumed that the exothermic peak is due to a physical process, namely the “cold” crystallization of 2-CEPP due to an increase in segmental mobility in the molecule after devitrification (the substance did not pass into the liquid phase). To test this hypothesis, 2-CEPP was heated to 200 °C and rapidly cooled, after which it was re-examined using small-angle X-ray scattering. On the X-ray diffraction pattern of the heated sample (Figure 6b), a group of peaks characteristic of the crystalline phase appeared, while the halo of the amorphous phase completely disappeared, which confirms the assumption of phosphazene crystallization.

When 2-CEPP is heated, two endothermic peaks are sequentially observed on the DSC curve with maxima at 240 and 260 °C, and no weight loss is observed on the TGA curve. Therefore, these peaks can be attributed to the melting of the substance. The presence of two peaks can be substantiated by the polymorphic nature of the formed crystals.

Above 270 °C, thermal destruction of the substance begins, and the TGA curve has the form of a characteristic step. In this case, the process of mass loss is accompanied by the absorption of heat. Accordingly, it can be assumed that this behavior of the substance is due to a specific chemical process, which begins at 270 °C and ends at 370 °C.

It is known that the pyrolysis of acids can be accompanied by a decarboxylation reaction and/or the formation of anhydrides. Based on the weight loss (25%), it can be concluded that the pyrolysis of 2-CEPP results in its complete decarboxylation. This was confirmed by solid-state ^13^C NMR spectroscopy. The spectrum of phosphazene heated to 370 °C (Figure 3B) lacks a peak at 168 ppm, which is characteristic of the carbon atoms of carboxyl groups, which is observed in the spectrum of the initial 2-CEPP (Figure 3A).

The product formed during pyrolysis turned out to be insoluble in any solvent. Since the decarboxylation of cinnamic acid is accompanied by the formation of styrene, it was suggested that the decarboxylation of 2-CEPP is also accompanied by the formation of vinyl bonds, which thermally polymerize and form a highly reticulated polymer. This assumption is confirmed by the presence of an exothermic polymerization peak in the region of 320–380 °C, as well as by the presence of carbon atom signals characteristic of carbochain units on the solid-state NMR spectrum in the region of 25–53 ppm (Figure 3B). The resulting polymer is thermally stable up to 500 °C.

**Figure 7:**
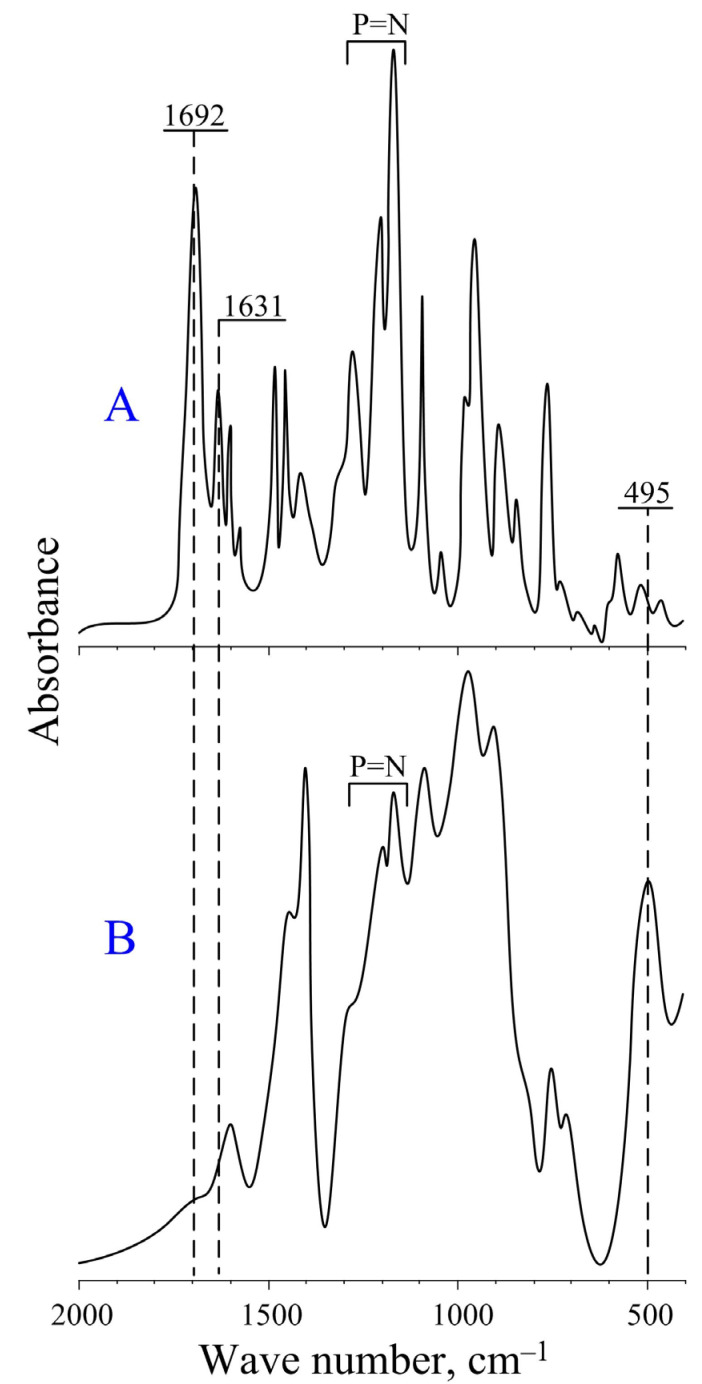
IR spectra of 2-CEPP (**A**) and 2-CEPP heated at 370 °C (**B**).

Additionally, this assumption was confirmed using IR-spectroscopy (Figure 7). On the IR spectrum of the 2-CEPP sample heated to 370 °C, the vibrational bands characteristic of the carbonyl group in the region of 1692 cm^−1^ and the carbon–carbon double bonds of cinnamic acid in the region of 1631 cm^−1^ disappear, and a peak appears for vibrations of the C-H bonds of polystyrene chains in the region of 495 cm^−1^. The presence of characteristic bands of phosphorus–nitrogen bond vibrations on the IR spectrum indicates the preservation of the phosphazene cycle during decarboxylation and polymerization.

It should be noted that another second-order phase transition was observed in the DSC curve at 480–500 °C, which can be explained by the devitrification of the highly reticulated polymer formed as a result of pyrolysis. Above this temperature, the polymer slowly undergoes further thermal degradation.

## 3. Materials and Methods

### 3.1. Materials

Malonic acid, pyridine, piperidine, hydrochloric acid, and deuterated dimethylsulfoxide (DMSO-*d_6_*) were products of Sigma Aldrich (Saint Louis, MO, USA).

### 3.2. Synthesis of 2-FPP

The synthesis and characterization of hexakis-2-(formylphenoxy)cyclotriphosphazene are presented in [45].

### 3.3. Synthesis of 2-CEPP

In a round-bottomed flask equipped with a magnetic stirrer, 2-FPP (2 g, 0.002 mol) and malonic acid (2.1 g, 0.02 mol) were dissolved in dry pyridine (2.5 mL). Then, one drop of piperidine was added to the reaction mixture. Synthesis lasted for 10 h at 115 °C. Then, the product was precipitated with an aqueous solution of hydrochloric acid (50 mL 36 wt.% HCl in 100 mL H_2_O). The resulting precipitate was filtered off, washed with distilled water, solid filtered, and dried. Compound 3 was obtained as a white solid. Yield: 2.4 g. ^31^P NMR (DMSO-*d_6_*, ppm): 9.0 (s). ^1^H NMR (DMSO-*d_6_*, ppm): 6.4–6.6 (=CH-COOH), 7.1–7.3 (CH_Ar_), 7.7–7.8 (-CH=CH-), 7.8–7.9 (CH_Ar_). ^13^C NMR (DMSO-d_6_, ppm): 119.9 (=CH-COOH), 121.2 (C_Ar_), 125.6 (C_Ar_), 125.8 (C_Ar_), 128.0 (C_Ar_), 131.1 (C_Ar_), 136.4 (-CH=CH-), 147.9 (C_Ar_), 168.0 (COOH).

### 3.4. Methods

^1^H, ^31^P, and ^13^C NMR spectra were recorded on a Bruker AV-400 spectrometer, (Bruker Corporation, Billerica, MA, USA). DMSO-*d_6_* was used as a solvent.

The solid-state ^13^C NMR spectrum was measured on a Varian NMR System NB 500 (Varian Inc., Palo Alto, CA, USA) at a frequency of 125.7 MHz. Before recording of the spectrum, the 2-CEPP sample was kept for 5 min in a heating cabinet at a temperature of 370 °C in a sealed ampoule filled with argon.

The mass spectrum was recorded on a Microflex LRF mass spectrometer (Bruker Daltonic GmbH, Leipzig, Germany).

IR spectra were measured on a Nicolet 380 spectrometer (Thermo Fisher Scientific, Waltham, MA, USA) in the spectral range of 4000–500 cm^−1^ with a wavenumber accuracy of 0.01 cm^−1^.

Elemental analysis was performed using an X-Max SDD Inca Energy-Dispersive Spectrometer for electron probe microanalysis (Oxford Instruments, Abingdon, UK).

Differential-scanning calorimetry (DSC) and thermogravimetric analysis (TGA) measurements were performed using the NETZSCH STA 449F1 (Erich NETZSCH GmbH & Co. Holding KG, Selb, Germany) instrument (10 °C min^−1^). Argon was used as a purge gas.

The small-angle X-ray scattering intensities were measured with the AMUR-K automated small-angle X-ray diffractometer (FRC Crystallography and Photonics, Moscow, Russia) with a linear position-sensitive detector (3300 channels) at a fixed wavelength λ = 0.1542 nm (CuKα line of a fine-focus tube, a pyrolytic graphite monochromator) and a Kratky collimation system. Before the study, the 2-CEPP sample was kept for 5 min in a heating cabinet at a temperature of 200 °C in a sealed and argon-filled ampoule.

Quantum-chemical calculation for the 2-CEPP molecule was carried out using the ab initio methods in the 6-311G^**^ basis set and the DFT-PBE0/6-311g** method, with geometry optimization of all parameters using the standard gradient method built into Firefly [46], which is partially based on the GAMESS (US) source code [47], for the approximation of an isolated molecule in the gas phase and a theoretical estimate of its acid strength. The calculations were performed for the ground state of the molecule under study. The total charge of the system was 0, and the multiplicity (M) was 1 (M = 2S + 1, total spin is 0, all electrons are paired). The MacMolPlt program [48] was used to visually represent the model of the molecule. The initial data were taken from the structure of the 2-FPP molecule [45]. Optimized bond lengths, bond angles, and atomic charges of the 2-CEPP molecule are presented in the Supplementary Materials.

## 4. Conclusions

In conclusion, in the present work, a method for the synthesis of hexakis-2-(β-carboxyethenylphenoxy)cyclotriphosphazene was proposed and its thermal behavior was studied. Using differential-scanning calorimetry and the method of small-angle X-ray scattering, it was found that phosphazene is amorphous at room temperature and crystallizes when it is heated to 185 °C, followed by rapid cooling. With the help of quantum chemistry methods, its structure was modeled, and the pK_a_ value was estimated. It has been established that phosphazene belongs to the weak acids and is soluble in industrial amine hardeners. The synthesized phosphazene is proposed to be used as a modifier of polymer binders for epoxy adhesive compositions for bonding metal parts in the aircraft and automotive industries and everyday life, since carboxyl groups in its composition will presumably increase adhesion to metal. In addition, due to the cooperative flame-retardant effect of the phosphorous and nitrogen atoms of the phosphazene cycle, epoxy compositions can have improved fire resistance. However, since 2-CEPP is insoluble in industrial epoxy resins at room temperature and only melts at 240–260 °C, more research is required to combine phosphazene with resins. Therefore, in the future, it is planned to conduct research on solutions combining 2-CEPP with resins, the selection of special additives, and the dilution of phosphazene with other hardeners, which make it possible to achieve homogeneous mixtures with epoxides.

## Figures and Tables

**Table 1:** Elemental composition of 2-CEFF (%).

Chemical Element	Actual Content		Theoretical Content	
	Weight	Atomic	Weight	Atomic
C	57.84	44.7	58.22	45
N	3.79	2.5	3.77	2.5
O	26.01	15.1	25.88	15
P	8.39	2.5	8.36	2.5
H	3.79	35.2	3.77	35

## Data Availability

The data presented in this study are available on request from the corresponding author.

## Supplementary Materials

The following supporting information can be downloaded at: https://www.mdpi.com/article/10.3390/molecules28186571/s1, Figure S1: Geometrical and electronic structure of the 2-CEPP molecule obtained ab initio (A) and by DFT (B); Table S1: Optimized bond lengths, bond angles, and charges on atoms of the 2-CEPP molecule obtained by the ab initio method; Table S2: Optimized bond lengths, bond angles, and charges on atoms of the 2-CEPP molecule obtained by the DFT method.

## Sample Availability

Samples of the compounds are available from the authors.
