# Supplementary material for: Hexakis-2-(β-carboxyethenylphenoxy)cyclotriphosphazene: Synthesis, Properties, Modeling Structure
Source: Molecules. 2023 Sep 11;28(18):6571. doi: 10.3390/molecules28186571 (PMC10534807; doi:10.3390/molecules28186571)
Supplement: Supplementary file 1 [file molecules-28-06571-s001.zip › molecules-2562038-supplementary.pdf]

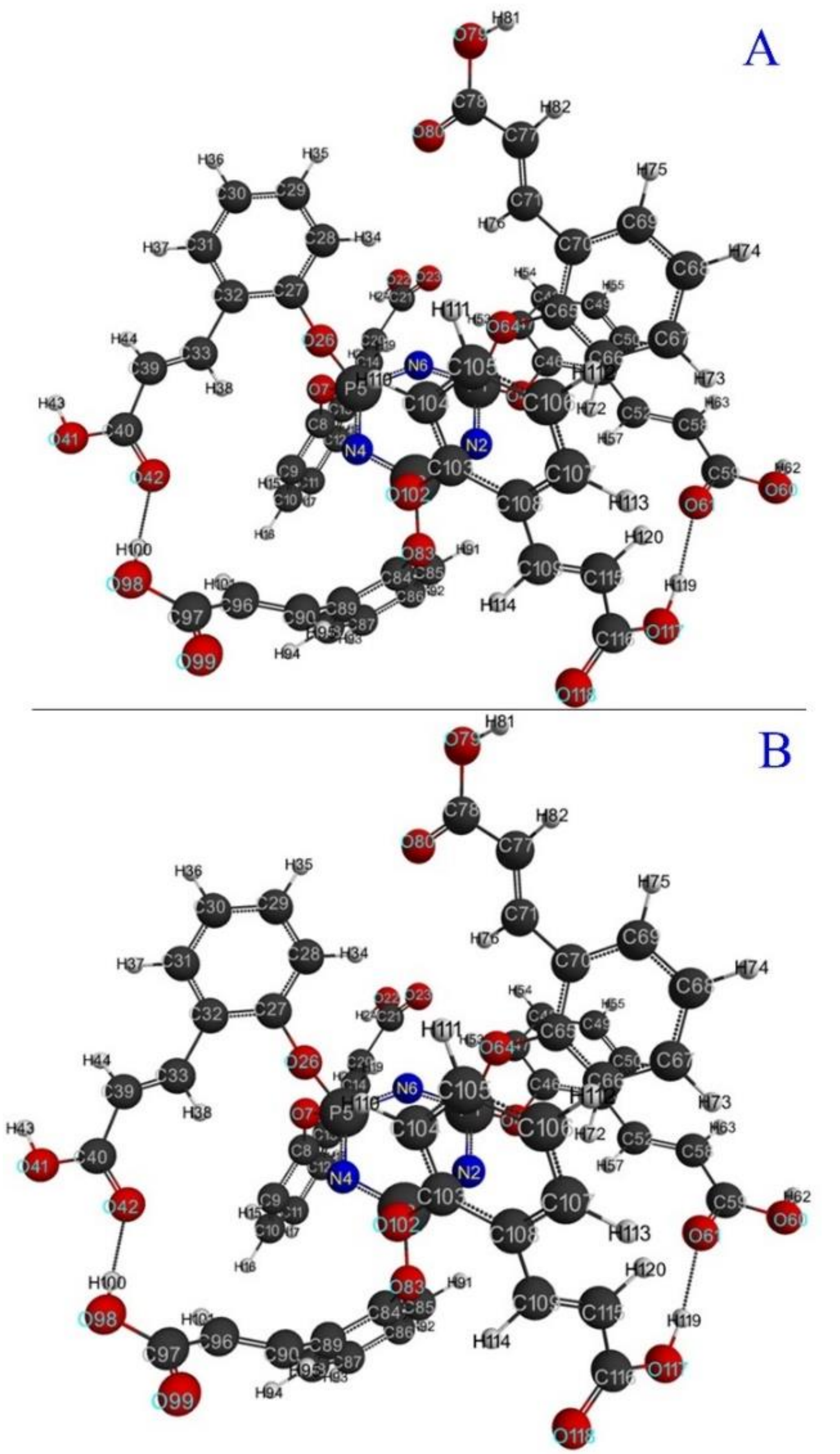

Figure S1. Geometrical and electronic structure of the 2-CEPP molecule obtained by ab initio (A) and DFT (B).

Table S1. Optimized bond lengths, bond angles, and charges on atoms of the 2-CEPP molecule obtained by the ab initio method.

Bond lengths	R, A	Bond angles	Degree	Atom	Charges on atoms of a molecule
N(2)-P(1)	1.57	P(1)-N(2)-P(3)	125	P(1)	+1.713
P(3)-N(2)	1.58	N(2)-P(3)-N(4)	114	N(2)	-1.012
N(4)-P(3)	1.58	P(3)-N(4)-P(5)	124	P(3)	+1.732
P(5)-N(4)	1.57	N(2)-P(1)-N(6)	116	N(4)	-0.982
N(6)-P(1)	1.57	N(4)-P(5)-O(7)	113	P(5)	+1.71
O(7)-P(5)	1.58	P(5)-O(7)-C(8)	132	N(6)	-0.99
C(8)-O(7)	1.37	O(7)-C(8)-C(9)	118	O(7)	-0.734
C(9)-C(8)	1.38	C(13)-C(8)-C(9)	122	C(8)	+0.271
C(10)-C(9)	1.38	C(8)-C(9)-C(10)	119	C(9)	-0.07
C(11)-C(10)	1.39	C(9)-C(10)-C(11)	120	C(10)	-0.081
C(12)-C(11)	1.38	C(13)-C(12)-C(11)	121	C(11)	-0.087
C(12)-C(13)	1.40	C(10)-C(11)-C(12)	120	C(12)	-0.061
C(13)-C(8)	1.39	C(8)-C(13)-C(12)	117	C(13)	-0.10
C(14)-C(13)	1.47	C(14)-C(13)-C(12)	122	C(14)	+0.03
H(15)-C(9)	1.07	O(7)-C(8)-C(13)	119	H(15)	+0.166
H(16)-C(10)	1.07	C(8)-C(13)-C(14)	121	H(16)	+0.106
H(17)-C(11)	1.07	C(8)-C(9)-H(15)	119	H(17)	+0.10
H(18)-C(12)	1.07	C(9)-C(10)-H(16)	120	H(18)	+0.098
H(19)-C(14)	1.07	C(10)-C(11)-H(17)	120	H(19)	+0.202
C(20)-C(14)	1.33	C(11)-C(12)-H(18)	119	C(20)	-0.337
C(21)-C(20)	1.49	C(13)-C(12)-H(18)	119	C(21)	+0.639
O(22)-C(21)	1.33	C(13)-C(14)-H(19)	117	O(22)	-0.39
O(23)-C(21)	1.18	C(13)-C(14)-C(20)	125	O(23)	-0.466
H(24)-O(22)	0.94	C(14)-C(20)-C(21)	120	H(24)	+0.262
H(25)-C(20)	1.08	C(20)-C(21)-O(22)	115	H(25)	+0.098
O(26)-P(5)	1.58	O(23)-C(21)-O(22)	120	O(26)	-0.714
C(27)-O(26)	1.37	C(20)-C(21)-O(23)	125	C(27)	+0.293
C(28)-C(27)	1.38	C(21)-O(22)-H(24)	112	C(28)	-0.095
C(29)-C(28)	1.38	C(14)-C(20)-H(25)	122	C(29)	-0.084
C(30)-C(29)	1.39	N(4)-P(5)-O(26)	107	C(30)	-0.09
C(31)-C(30)	1.38	P(5)-O(26)-C(27)	130	C(31)	-0.048
C(31)-C(32)	1.39	O(26)-C(27)-C(28)	119	C(32)	-0.085
C(32)-C(27)	1.39	C(32)-C(27)-C(28)	122	C(33)	+0.021
C(33)-C(32)	1.47	C(27)-C(28)-C(29)	119	H(34)	+0.158
H(34)-C(28)	1.07	C(28)-C(29)-C(30)	120	H(35)	+0.139
H(35)-C(29)	1.07	C(32)-C(31)-C(30)	121	H(36)	+0.101
H(36)-C(30)	1.07	C(29)-C(30)-C(31)	120	H(37)	+0.093
H(37)-C(31)	1.07	C(27)-C(32)-C(31)	117	H(38)	+0.171
H(38)-C(33)	1.07	C(33)-C(32)-C(31)	123	C(39)	-0.331
C(39)-C(33)	1.33	O(26)-C(27)-C(32)	119	C(40)	+0.669
C(40)-C(39)	1.48	C(27)-C(32)-C(33)	120	O(41)	-0.382
O(41)-C(40)	1.33	C(27)-C(28)-H(34)	120	O(42)	-0.524
O(42)-C(40)	1.19	C(28)-C(29)-H(35)	119	H(43)	+0.267
O(42)-H(100)	1.96	C(29)-C(30)-H(36)	120	H(44)	+0.101
H(43)-O(41)	0.94	C(30)-C(31)-H(37)	119	O(45)	-0.716
H(44)-C(39)	1.08	C(32)-C(31)-H(37)	119	C(46)	+0.301
O(45)-P(1)	1.58	C(32)-C(33)-H(38)	116	C(47)	-0.136
C(46)-O(45)	1.37	C(32)-C(33)-C(39)	127	C(48)	-0.08
C(46)-C(51)	1.39	C(33)-C(39)-C(40)	120	C(49)	-0.092

C(47)-C(46)	1.38	C(39)-C(40)-O(41)	116	C(50)	-0.04
C(48)-C(47)	1.38	O(42)-C(40)-O(41)	120	C(51)	-0.122
C(49)-C(48)	1.39	C(39)-C(40)-O(42)	124	C(52)	+0.034
C(50)-C(49)	1.38	C(40)-O(41)-H(43)	112	H(53)	+0.189
C(51)-C(50)	1.39	C(33)-C(39)-H(44)	122	H(54)	+0.137
C(52)-C(51)	1.47	N(2)-P(1)-O(45)	106	H(55)	+0.102
H(53)-C(47)	1.07	C(51)-C(46)-O(45)	117	H(56)	+0.093
H(54)-C(48)	1.07	P(1)-O(45)-C(46)	132	H(57)	+0.173
H(55)-C(49)	1.07	C(50)-C(51)-C(46)	117	C(58)	-0.33
H(56)-C(50)	1.07	C(52)-C(51)-C(46)	119	C(59)	+0.669
H(57)-C(52)	1.07	O(45)-C(46)-C(47)	121	O(60)	-0.38
C(58)-C(52)	1.33	C(51)-C(46)-C(47)	122	O(61)	-0.526
C(59)-C(58)	1.48	C(46)-C(47)-C(48)	119	H(62)	+0.268
O(60)-C(59)	1.33	C(47)-C(48)-C(49)	120	H(63)	+0.10
O(61)-C(59)	1.19	C(48)-C(49)-C(50)	120	O(64)	-0.729
O(61)-H(119)	1.98	C(49)-C(50)-C(51)	121	C(65)	+0.34
H(62)-O(60)	0.94	C(50)-C(51)-C(52)	123	C(66)	-0.151
H(63)-C(58)	1.08	C(46)-C(47)-H(53)	121	C(67)	-0.075
O(64)-P(1)	1.59	C(47)-C(48)-H(54)	119	C(68)	-0.098
C(65)-O(64)	1.37	C(48)-C(49)-H(55)	120	C(69)	-0.049
C(65)-C(70)	1.40	C(49)-C(50)-H(56)	119	C(70)	-0.143
C(66)-C(65)	1.38	C(51)-C(52)-H(57)	116	C(71)	+0.047
C(67)-C(66)	1.38	C(51)-C(52)-C(58)	128	H(72)	+0.196
C(68)-C(67)	1.38	C(52)-C(58)-C(59)	119	H(73)	+0.114
C(69)-C(68)	1.38	C(58)-C(59)-O(60)	116	H(74)	+0.10
C(70)-C(69)	1.39	O(61)-C(59)-O(60)	120	H(75)	+0.096
C(71)-C(70)	1.47	C(58)-C(59)-O(61)	124	H(76)	+0.178
H(72)-C(66)	1.07	C(59)-O(60)-H(62)	112	C(77)	-0.339
H(73)-C(67)	1.07	C(52)-C(58)-H(63)	123	C(78)	+0.64
H(74)-C(68)	1.07	N(2)-P(1)-O(64)	113	O(79)	-0.389
H(75)-C(69)	1.07	C(70)-C(65)-O(64)	116	O(80)	-0.464
H(76)-C(71)	1.07	P(1)-O(64)-C(65)	134	H(81)	+0.261
C(77)-C(71)	1.33	C(69)-C(70)-C(65)	118	H(82)	+0.098
C(78)-C(77)	1.49	C(71)-C(70)-C(65)	119	O(83)	-0.711
O(79)-C(78)	1.33	O(64)-C(65)-C(66)	122	C(84)	+0.297
O(80)-C(78)	1.18	C(70)-C(65)-C(66)	122	C(85)	-0.079
H(81)-O(79)	0.94	C(65)-C(66)-C(67)	119	C(86)	-0.078
H(82)-C(77)	1.08	C(66)-C(67)-C(68)	121	C(87)	-0.089
O(83)-P(3)	1.57	C(67)-C(68)-C(69)	120	C(88)	-0.075
C(84)-O(83)	1.37	C(68)-C(69)-C(70)	121	C(89)	-0.066
C(85)-C(84)	1.38	C(69)-C(70)-C(71)	123	C(90)	-0.023
C(86)-C(85)	1.38	C(65)-C(66)-H(72)	120	H(91)	+0.123
C(87)-C(86)	1.38	C(66)-C(67)-H(73)	119	H(92)	+0.102
C(88)-C(87)	1.38	C(67)-C(68)-H(74)	120	H(93)	+0.097
C(88)-C(89)	1.39	C(68)-C(69)-H(75)	119	H(94)	+0.106
C(89)-C(84)	1.39	C(70)-C(71)-H(76)	116	H(95)	+0.164
C(90)-C(89)	1.48	C(70)-C(71)-C(77)	127	C(96)	-0.281
H(91)-C(85)	1.07	C(71)-C(77)-C(78)	120	C(97)	+0.593
H(92)-C(86)	1.07	C(77)-C(78)-O(79)	115	O(98)	-0.41
H(93)-C(87)	1.08	O(80)-C(78)-O(79)	120	O(99)	-0.453
H(94)-C(88)	1.07	C(77)-C(78)-O(80)	125	H(100)	+0.298
H(95)-C(90)	1.08	C(78)-O(79)-H(81)	112	H(101)	+0.114
C(96)-C(90)	1.32	C(71)-C(77)-H(82)	122	O(102)	-0.701

C(97)-C(96)	1.50	N(2)-P(3)-O(83)	112	C(103)	+0.293
O(98)-C(97)	1.32	P(3)-O(83)-C(84)	130	C(104)	-0.081
O(99)-C(97)	1.18	O(83)-C(84)-C(85)	119	C(105)	-0.082
H(100)-O(98)	0.95	C(89)-C(84)-C(85)	122	C(106)	-0.081
H(101)-C(96)	1.08	C(84)-C(85)-C(86)	119	C(107)	-0.067
O(102)-P(3)	1.58	C(85)-C(86)-C(87)	120	C(108)	-0.07
C(103)-O(102)	1.38	C(89)-C(88)-C(87)	121	C(109)	-0.034
C(104)-C(103)	1.38	C(86)-C(87)-C(88)	120	H(110)	+0.123
C(105)-C(104)	1.38	C(84)-C(89)-C(88)	117	H(111)	+0.103
C(106)-C(105)	1.38	C(90)-C(89)-C(88)	122	H(112)	+0.098
C(107)-C(106)	1.38	O(83)-C(84)-C(89)	118	H(113)	+0.104
C(107)-C(108)	1.39	C(84)-C(89)-C(90)	121	H(114)	+0.169
C(108)-C(103)	1.39	C(84)-C(85)-H(91)	119	C(115)	-0.302
C(109)-C(108)	1.48	C(85)-C(86)-H(92)	120	C(116)	+0.599
H(110)-C(104)	1.07	C(86)-C(87)-H(93)	120	O(117)	-0.412
H(111)-C(105)	1.07	C(87)-C(88)-H(94)	120	O(118)	-0.454
H(112)-C(106)	1.07	C(89)-C(88)-H(94)	119	H(119)	+0.297
H(113)-C(107)	1.07	C(89)-C(90)-H(95)	117	H(120)	+0.118
H(114)-C(109)	1.08	C(89)-C(90)-C(96)	125		
C(115)-C(109)	1.32	C(90)-C(96)-C(97)	120		
C(116)-C(115)	1.49	C(96)-C(97)-O(98)	115		
O(117)-C(116)	1.32	O(99)-C(97)-O(98)	121		
O(118)-C(116)	1.18	C(96)-C(97)-O(99)	123		
H(119)-O(117)	0.95	C(97)-O(98)-H(100)	113		
H(120)-C(115)	1.08	C(90)-C(96)-H(101)	122		
		N(2)-P(3)-O(102)	109		
		P(3)-O(102)-C(103)	129		
		O(102)-C(103)-C(104)	118		
		C(108)-C(103)-C(104)	122		
		C(103)-C(104)-C(105)	119		
		C(104)-C(105)-C(106)	120		
		C(108)-C(107)-C(106)	121		
		C(105)-C(106)-C(107)	120		
		C(103)-C(108)-C(107)	117		
		C(109)-C(108)-C(107)	122		
		O(102)-C(103)-C(108)	120		
		C(103)-C(108)-C(109)	121		
		C(103)-C(104)-H(110)	119		
		C(104)-C(105)-H(111)	120		
		C(105)-C(106)-H(112)	120		
		C(106)-C(107)-H(113)	120		
		C(108)-C(107)-H(113)	119		
		C(108)-C(109)-H(114)	117		
		C(108)-C(109)-C(115)	125		
		C(109)-C(115)-C(116)	120		
		C(115)-C(116)-O(117)	116		
		O(118)-C(116)-O(117)	121		
		C(115)-C(116)-O(118)	123		
		C(116)-O(117)-H(119)	114		
		C(109)-C(115)-H(120)	122		

Table S2. Optimized bond lengths, bond angles, and charges on atoms of the 2-CEPP molecule obtained by DFT method.

Bond lengths	R, A	Bond angles	Degree	Atom	Charges on atoms of a molecule
N(2)-P(1)	1.58	P(1)-N(2)-P(3)	122	P(1)	+1.318
P(3)-N(2)	1.60	N(2)-P(3)-N(4)	116	N(2)	-0.785
N(4)-P(3)	1.60	P(3)-N(4)-P(5)	122	P(3)	+1.322
P(5)-N(4)	1.58	N(2)-P(1)-N(6)	118	N(4)	-0.752
N(6)-P(1)	1.59	N(4)-P(5)-O(7)	113	P(5)	+1.326
O(7)-P(5)	1.60	P(5)-O(7)-C(8)	128	N(6)	-0.769
C(8)-O(7)	1.38	O(7)-C(8)-C(9)	118	O(7)	-0.579
C(9)-C(8)	1.39	C(13)-C(8)-C(9)	122	C(8)	+0.163
C(10)-C(9)	1.39	C(8)-C(9)-C(10)	119	C(9)	-0.046
C(11)-C(10)	1.39	C(9)-C(10)-C(11)	120	C(10)	-0.108
C(12)-C(11)	1.38	C(13)-C(12)-C(11)	122	C(11)	-0.087
C(12)-C(13)	1.40	C(10)-C(11)-C(12)	120	C(12)	-0.048
C(13)-C(8)	1.40	C(8)-C(13)-C(12)	117	C(13)	-0.089
C(14)-C(13)	1.46	C(14)-C(13)-C(12)	123	C(14)	-0.007
H(15)-C(9)	1.08	O(7)-C(8)-C(13)	119	H(15)	+0.179
H(16)-C(10)	1.08	C(8)-C(13)-C(14)	120	H(16)	+0.11
H(17)-C(11)	1.08	C(8)-C(9)-H(15)	119	H(17)	+0.104
H(18)-C(12)	1.08	C(9)-C(10)-H(16)	119	H(18)	+0.099
H(19)-C(14)	1.09	C(10)-C(11)-H(17)	120	H(19)	+0.189
C(20)-C(14)	1.34	C(11)-C(12)-H(18)	119	C(20)	-0.298
C(21)-C(20)	1.48	C(13)-C(12)-H(18)	119	C(21)	+0.473
O(22)-C(21)	1.35	C(13)-C(14)-H(19)	117	O(22)	-0.313
O(23)-C(21)	1.20	C(13)-C(14)-C(20)	126	O(23)	-0.368
H(24)-O(22)	0.96	C(14)-C(20)-C(21)	120	H(24)	+0.249
H(25)-C(20)	1.09	C(20)-C(21)-O(22)	116	H(25)	+0.108
O(26)-P(5)	1.61	O(23)-C(21)-O(22)	120	O(26)	-0.555
C(27)-O(26)	1.38	C(20)-C(21)-O(23)	125	C(27)	+0.17
C(27)-C(32)	1.40	C(21)-O(22)-H(24)	110	C(28)	-0.066
C(28)-C(27)	1.38	C(14)-C(20)-H(25)	121	C(29)	-0.096
C(29)-C(28)	1.39	N(4)-P(5)-O(26)	106	C(30)	-0.095
C(30)-C(29)	1.39	C(32)-C(27)-O(26)	119	C(31)	-0.033
C(31)-C(30)	1.38	P(5)-O(26)-C(27)	127	C(32)	-0.065
C(32)-C(31)	1.40	C(31)-C(32)-C(27)	117	C(33)	-0.037
C(33)-C(32)	1.45	C(33)-C(32)-C(27)	119	H(34)	+0.16
H(34)-C(28)	1.08	O(26)-C(27)-C(28)	119	H(35)	+0.136
H(35)-C(29)	1.08	C(32)-C(27)-C(28)	122	H(36)	+0.107
H(36)-C(30)	1.08	C(27)-C(28)-C(29)	119	H(37)	+0.097
H(37)-C(31)	1.08	C(28)-C(29)-C(30)	120	H(38)	+0.175
H(38)-C(33)	1.09	C(29)-C(30)-C(31)	120	C(39)	-0.28
C(39)-C(33)	1.34	C(30)-C(31)-C(32)	121	C(40)	+0.498
C(40)-C(39)	1.47	C(31)-C(32)-C(33)	124	O(41)	-0.302
O(41)-C(40)	1.34	C(27)-C(28)-H(34)	120	O(42)	-0.427
O(42)-C(40)	1.21	C(28)-C(29)-H(35)	118	H(43)	+0.256
O(42)-H(100)	1.84	C(29)-C(30)-H(36)	120	H(44)	+0.114
H(43)-O(41)	0.96	C(30)-C(31)-H(37)	119	O(45)	-0.553
H(44)-C(39)	1.09	C(32)-C(33)-H(38)	116	C(46)	+0.178
O(45)-P(1)	1.60	C(32)-C(33)-C(39)	128	C(47)	-0.097
C(46)-O(45)	1.38	C(33)-C(39)-C(40)	119	C(48)	-0.092
C(46)-C(51)	1.41	C(39)-C(40)-O(41)	116	C(49)	-0.096

C(47)-C(46)	1.38	O(42)-C(40)-O(41)	119	C(50)	-0.03
C(48)-C(47)	1.39	C(39)-C(40)-O(42)	124	C(51)	-0.109
C(49)-C(48)	1.39	C(40)-O(41)-H(43)	110	C(52)	-0.011
C(50)-C(49)	1.38	C(33)-C(39)-H(44)	122	H(53)	+0.186
C(51)-C(50)	1.40	N(2)-P(1)-O(45)	104	H(54)	+0.137
C(52)-C(51)	1.45	C(51)-C(46)-O(45)	117	H(55)	+0.108
H(53)-C(47)	1.08	P(1)-O(45)-C(46)	128	H(56)	+0.097
H(54)-C(48)	1.08	C(50)-C(51)-C(46)	117	H(57)	+0.17
H(55)-C(49)	1.08	C(52)-C(51)-C(46)	119	C(58)	-0.282
H(56)-C(50)	1.08	O(45)-C(46)-C(47)	121	C(59)	+0.497
H(57)-C(52)	1.09	C(51)-C(46)-C(47)	122	O(60)	-0.30
C(58)-C(52)	1.34	C(46)-C(47)-C(48)	119	O(61)	-0.431
C(59)-C(58)	1.47	C(47)-C(48)-C(49)	120	H(62)	+0.256
O(60)-C(59)	1.34	C(48)-C(49)-C(50)	120	H(63)	+0.114
O(61)-C(59)	1.21	C(49)-C(50)-C(51)	121	O(64)	-0.57
O(61)-H(119)	1.84	C(50)-C(51)-C(52)	124	C(65)	+0.258
H(62)-O(60)	0.96	C(46)-C(47)-H(53)	121	C(66)	-0.158
H(63)-C(58)	1.09	C(47)-C(48)-H(54)	118	C(67)	-0.085
O(64)-P(1)	1.62	C(48)-C(49)-H(55)	120	C(68)	-0.10
C(65)-O(64)	1.37	C(49)-C(50)-H(56)	120	C(69)	-0.036
C(65)-C(70)	1.41	C(51)-C(52)-H(57)	116	C(70)	-0.147
C(66)-C(65)	1.39	C(51)-C(52)-C(58)	129	C(71)	-0.004
C(67)-C(66)	1.39	C(52)-C(58)-C(59)	119	H(72)	+0.215
C(68)-C(67)	1.39	C(58)-C(59)-O(60)	117	H(73)	+0.122
C(69)-C(68)	1.38	O(61)-C(59)-O(60)	119	H(74)	+0.104
C(70)-C(69)	1.40	C(58)-C(59)-O(61)	124	H(75)	+0.098
C(71)-C(70)	1.45	C(59)-O(60)-H(62)	110	H(76)	+0.183
H(72)-C(66)	1.08	C(52)-C(58)-H(63)	123	C(77)	-0.302
H(73)-C(67)	1.08	N(2)-P(1)-O(64)	113	C(78)	+0.47
H(74)-C(68)	1.08	C(70)-C(65)-O(64)	116	O(79)	-0.311
H(75)-C(69)	1.08	P(1)-O(64)-C(65)	130	O(80)	-0.364
H(76)-C(71)	1.09	C(69)-C(70)-C(65)	117	H(81)	+0.248
C(77)-C(71)	1.34	C(71)-C(70)-C(65)	119	H(82)	+0.108
C(78)-C(77)	1.48	O(64)-C(65)-C(66)	123	O(83)	-0.549
O(79)-C(78)	1.35	C(70)-C(65)-C(66)	122	C(84)	+0.168
O(80)-C(78)	1.20	C(65)-C(66)-C(67)	119	C(85)	-0.047
H(81)-O(79)	0.96	C(66)-C(67)-C(68)	121	C(86)	-0.092
H(82)-C(77)	1.09	C(67)-C(68)-C(69)	120	C(87)	-0.087
O(83)-P(3)	1.60	C(68)-C(69)-C(70)	121	C(88)	-0.083
C(84)-O(83)	1.38	C(69)-C(70)-C(71)	124	C(89)	-0.058
C(84)-C(89)	1.40	C(65)-C(66)-H(72)	120	C(90)	-0.025
C(85)-C(84)	1.38	C(66)-C(67)-H(73)	119	H(91)	+0.132
C(86)-C(85)	1.39	C(67)-C(68)-H(74)	120	H(92)	+0.108
C(87)-C(86)	1.39	C(68)-C(69)-H(75)	120	H(93)	+0.101
C(88)-C(87)	1.39	C(70)-C(71)-H(76)	116	H(94)	+0.111
C(89)-C(88)	1.40	C(70)-C(71)-C(77)	128	H(95)	+0.164
C(90)-C(89)	1.46	C(71)-C(77)-C(78)	119	C(96)	-0.278
H(91)-C(85)	1.08	C(77)-C(78)-O(79)	116	C(97)	+0.423
H(92)-C(86)	1.08	O(80)-C(78)-O(79)	120	O(98)	-0.328
H(93)-C(87)	1.08	C(77)-C(78)-O(80)	125	O(99)	-0.354
H(94)-C(88)	1.09	C(78)-O(79)-H(81)	110	H(100)	+0.268
H(95)-C(90)	1.09	C(71)-C(77)-H(82)	122	H(101)	+0.123
C(96)-C(90)	1.34	N(2)-P(3)-O(83)	112	O(102)	-0.536

C(97)-C(96)	1.49	C(89)-C(84)-O(83)	118	C(103)	+0.176
O(98)-C(97)	1.34	P(3)-O(83)-C(84)	125	C(104)	-0.057
O(99)-C(97)	1.20	C(88)-C(89)-C(84)	117	C(105)	-0.092
H(100)-O(98)	0.97	C(90)-C(89)-C(84)	121	C(106)	-0.085
H(101)-C(96)	1.09	O(83)-C(84)-C(85)	119	C(107)	-0.063
O(102)-P(3)	1.60	C(89)-C(84)-C(85)	122	C(108)	-0.075
C(103)-O(102)	1.38	C(84)-C(85)-C(86)	119	C(109)	-0.047
C(103)-C(108)	1.40	C(85)-C(86)-C(87)	120	H(110)	+0.126
C(104)-C(103)	1.39	C(86)-C(87)-C(88)	120	H(111)	+0.109
C(105)-C(104)	1.39	C(87)-C(88)-C(89)	121	H(112)	+0.103
C(106)-C(105)	1.39	C(88)-C(89)-C(90)	122	H(113)	+0.11
C(107)-C(106)	1.39	C(84)-C(85)-H(91)	119	H(114)	+0.175
C(108)-C(107)	1.40	C(85)-C(86)-H(92)	120	C(115)	-0.306
C(109)-C(108)	1.46	C(86)-C(87)-H(93)	120	C(116)	+0.433
H(110)-C(104)	1.08	C(87)-C(88)-H(94)	120	O(117)	-0.33
H(111)-C(105)	1.08	C(89)-C(90)-H(95)	117	O(118)	-0.357
H(112)-C(106)	1.08	C(89)-C(90)-C(96)	125	H(119)	+0.269
H(113)-C(107)	1.08	C(90)-C(96)-C(97)	120	H(120)	+0.134
H(114)-C(109)	1.09	C(96)-C(97)-O(98)	116		
C(115)-C(109)	1.34	O(99)-C(97)-O(98)	121		
C(116)-C(115)	1.49	C(96)-C(97)-O(99)	123		
O(117)-C(116)	1.34	C(97)-O(98)-H(100)	111		
O(118)-C(116)	1.20	C(90)-C(96)-H(101)	121		
H(119)-O(117)	0.97	N(2)-P(3)-O(102)	109		
H(120)-C(115)	1.09	C(108)-C(103)-O(102)	120		
		P(3)-O(102)-C(103)	126		
		C(107)-C(108)-C(103)	117		
		C(109)-C(108)-C(103)	121		
		O(102)-C(103)-C(104)	117		
		C(108)-C(103)-C(104)	122		
		C(103)-C(104)-C(105)	120		
		C(104)-C(105)-C(106)	120		
		C(105)-C(106)-C(107)	120		
		C(106)-C(107)-C(108)	122		
		C(107)-C(108)-C(109)	122		
		C(103)-C(104)-H(110)	119		
		C(104)-C(105)-H(111)	120		
		C(105)-C(106)-H(112)	120		
		C(106)-C(107)-H(113)	120		
		C(108)-C(109)-H(114)	118		
		C(108)-C(109)-C(115)	125		
		C(109)-C(115)-C(116)	120		
		C(115)-C(116)-O(117)	116		
		O(118)-C(116)-O(117)	121		
		C(115)-C(116)-O(118)	123		
		C(116)-O(117)-H(119)	112		
		C(109)-C(115)-H(120)	122		
